# A new celll model of glutaric acidemia type I and the toxicity research

**DOI:** 10.1186/1687-9856-2013-S1-P183

**Published:** 2013-10-03

**Authors:** Cai Zhang, Xiaoping Luo

**Affiliations:** 1 Tongji Hospital, Wuhan, China

## 

Glutaric acidemia (GA-1) is an autosomal recessive disorder of lysine, hydroxylysine,and tryptophan metabolism caused by deficiency of glutaryl-CoA dehydrogenase. It results in the accumulation of 3-hydroxyglutaric and glutaric acid. Affected patients can present with brain atrophy and macrocephaly and with acute dystonia secondary to striatal degeneration in most cases triggered by an intercurrent childhood infection with fever between 6 and 18 months of age. The most common animal model of GA-1 is GCDH deficient mouse. We are trying to establish a new cell model of GA-1 to study the mechanism of neurotoxicity and for the future establishment of the new animal model.

Three Gcdh shRNA sequences were recombinated to the lentivirus vector carring the GFP reporter gene. And the Gcdh-shRNA vector with the highest knock-down efficiecy was transfected into the primary striatial neuron cells. Real-time PCR and Western Bot showed the knock-down efficiecy was about 65%. And compared with the control group, the cells transfected with Gcdh shRNA vector shrinked obviously and gathered together, the neurites disappeared, and cell debris increased significantly. Hoechst staining of the nucleus showed the increased apoptotic rate in the knock-down group. MTT indicated the decreased mitochondrial function after Gcdh shRNA interference. These results suggest that Gcdh shRNA interference in rat primary stiatial neuron cells could increase the apoptotic rate, which could due to the activation of mitochondria mediated apoptosis, tiggered by the dysfunction of the mitochondria.

